# Daily Functionality of People with Low Vision: The Impact of Visual Acuity, Depression, and Life Orientation—A Cross-Sectional Study

**DOI:** 10.1155/2024/4366572

**Published:** 2024-02-26

**Authors:** Mara Gkioka, Stavroula Almpanidou, Niki Lioti, Diamantis Almaliotis, Vasileios Karampatakis

**Affiliations:** Laboratory of Experimental Ophthalmology, School of Medicine, Aristotle University of Thessaloniki, Thessaloniki, Greece

## Abstract

**Background:**

Low vision (LV) has a significant negative impact on the activities of daily life as well as on the psychological health of patients.

**Objectives:**

The objective of this study is to investigate psychological, clinical, and demographic factors that may impact the daily functionality of patients with LV.

**Methods:**

A convenience sample of 53 patients, meeting the WHO criteria for LV, was recruited. Questionnaires on daily functionality, depression, and life orientation (in terms of optimism/pessimism) were administered along with a semistructured personal interview. *Key Findings*. The main results revealed a significant negative correlation between daily functionality and depression (*r* = −0.423, *p* < 0.001). Conversely, there is a positive correlation between daily functionality and visual acuity (*r* = 0.415, *p* < 0.001), while years since diagnosis were negatively correlated with depression (*r* = −0.345, *p* < 0.001). Depression seems to be a moderate predictor of a person's daily functionality (*β* = −0.389, *p* < 0.002), followed by visual acuity (*β* = −0.344, *p* = 0.006), explaining the 31.1% of the total variance.

**Conclusions:**

The study supports a correlation between daily functionality and both depression and visual acuity. Optimism as a personality characteristic did not factor into the prediction model for daily functionality, but it showed a strong correlation with lower levels of depressive symptoms. This highlights the potential for developing coping strategies for chronic disease management. *Recommendations*. The study could serve as a useful guide and may urge clinicians to pay attention to the psychological evaluation of these patients, supporting their unique emotional needs. Mental health professionals can use patients' positive resources to provide appropriate counseling and embrace the coping skills that encourage their engagement in activities of daily life.

## 1. Introduction

Vision is a fundamental sense for an individual's daily functionality, while being essential for optimal orientation and social life, affecting also physical and emotional well-being [1]. Low vision (LV)—according to the World Health Organization (WHO) classification system—is defined as “visual acuity less than 6/18 and equal to or better than 3/60 in the better eye with best correction” [2]. LV causes severe and chronic impairment, altering the ability of patients to successfully perform various daily activities [3].

Individuals with LV are more likely to experience limitations in daily functionality, independence, mobility [4, 5], and educational achievements, having also increased risk of falls, fractures, injuries, poor mental health, and social isolation [6], and the quality of life (QoL) is generally poor [1]. More specifically, LV affects patients' independent lives and affects many daily activities such as reading, socializing, engaging in hobbies [7], eating, dressing, shopping, financial and medication management, and driving [7–9]. LV is significantly associated with self-reported difficulty with walking or climbing steps [10] and a higher risk of increased falls [11] due to the related changes in visual acuity, visual fields, depth perception, contrast sensitivity, and poor dark adaptation [12–15]. LV has been also associated with an increased risk of fractures in multiple studies, while in one study, reversing visual impairment from cataract protects 67% from fractures [16]. Regarding poor QoL, there is strong evidence that it is related to the severity of LV disease (glaucoma, cataract, age-related macular degeneration, and strabismus) [17–22], while ocular diseases that primarily affect peripheral vision such as glaucoma seem to have a greater impact on QoL [23]. However, a systematic literature review highlighted that both types of vision loss (central and peripheral) were associated with similar levels of QoL decrease, which “might be a function of the pathology of diseases” [24]. A recent meta-analysis concluded that LV rehabilitation interventions may improve the QoL related to vision, mainly through psychological therapies and methods of enhancing vision [25].

Patient's self-reported QoL and daily functionality are critical to fully understanding the impact of ocular diseases and treatment options [26]. The need for patient-reported information has led to the development of various questionnaires assessing psychometric constructs. However, a construct such as “visual functioning” is often confused with “vision-specific QoL” [27]. “Vision-related QoL” is estimated as “a complex trait that encompasses visual functioning, symptoms, emotional well-being, social relationships, concerns, and convenience as they are affected by vision” [28]. Vision-related QoL has been extensively assessed with relevant questionnaires such as the Low Vision Quality-of-Life Questionnaire (LVQOL) [29] and the NEI-Visual Functioning Questionnaire 25 (NEI-VFQ-25) [30]. On the other hand, “visual functioning” is commonly assessed using several questionnaires such as Visual Function-14 (VF-14) [31] and the new Life for Low Vision Questionnaire (LIFE4LVQ) [32], which measure the impact of LV on activities of daily living (ADL). These questionnaires specifically measure limitations in daily activities (disability and/or dependence) associated with vision-dependent tasks such as reading, driving, and shopping, and they have been used to evaluate outcomes of rehabilitation services or even clinical trials [33–36].

The impact of LV extends to psychological and cognitive health, as those with LV have an increased risk of experiencing depression, anxiety, and other psychological conditions when compared to individuals with normal vision [3]. Additionally, cognitive functions, including learning, memory, information processing, and spatial representation, are also affected [37, 38]. In some cases (LV due to age-related macular degeneration (AMD)), there is a high incidence of cognitive impairment and an increased risk of developing dementia [39]. Furthermore, the loss of cognitively stimulating activities, such as reading, due to vision impairment can lead to a decline in other cognitive abilities [40]. Moreover, the reduced spatial orientation results in a loss of independence and control over one's environment, which can evoke feelings of anxiety and low self-esteem [41]. A wide range of emotional reactions are often observed such as feelings of inferiority and isolation, denial, anxiety, and depression [42]. More specifically, visual impairment is directly linked to depression and can even double the risk of developing depression [43]. The prevalence of depression among older individuals with impaired vision ranges from about 14% to 63% [44, 45]. Besides, the inability to accept their condition may lead to decreased social participation [46] and difficulty in engaging in leisure activities and in maintaining personal relationships [47]. There are also cases where the social burden is increased due to attitudes of compassion and overprotection from those around them [48, 49]. Several studies have emphasized the danger of loneliness not just for the elderly with LV [50] but also for younger and middle-aged individuals [51]. However, the implementation of strategies that can reduce loneliness could improve life satisfaction among visually impaired individuals [52]. There is also growing interest in the role of various factors such as positive attitude and personality characteristics in helping patients to adapt to their LV status [53]. The severity of the visual impairment, the “structure” of patients' life before the onset of the problem, and their general attitude and thoughts towards life, optimistic or pessimistic, may alter the degree of adaptation of the LV patients to their current visual status and influence their emotional and psychological variables [54].

Given all the above, the present study is aimed at investigating psychological, clinical, and demographic factors that may impact the daily functionality of patients with LV as assessed with the novel LIFE4LVQ [32].

## 2. Methods

### 2.1. Design

This is a quantitative, cross-sectional study design. The current study adheres to EQUATOR guidelines for reporting research using the “Strengthening the Reporting of Observational Studies in Epidemiology” (STROBE) checklist [55]. According to the research hypothesis, the impact of the demographics (age, gender, and education), the clinical evaluation (visual acuity, years of diagnosis, and systemic comorbidities), the psychological assessment (CES and LOT-R), and the subjective perspective of general health and general vision (independent variables) on the daily functionality of people with low vision (LIFE4LVQ) (dependent variable) will be explored.

### 2.2. Setting and Participants

This study was conducted between May 2019 and December 2021. Participants with visual impairment due to various ocular diseases were recruited at the outpatient unit for patients with LV at the School of Medicine of Aristotle University of Thessaloniki. To be eligible, participants had to be at least 18 years old and meet the WHO criteria for LV. Patients who fulfilled the eligibility criteria were informed about the aim of the study and signed a written consent. All patients participated voluntarily. Individuals who were cognitively impaired as defined by the Mini-Mental State Examination [56] were excluded. The convenience sample was comprised of fifty-three (53) LV individuals. Through an in-person interview, participants completed a series of medical examinations regarding the visual function and then completed demographic data and self-reported questionnaires about daily living, mood, a personality scale, and questions about the subjective perception of general health and vision. The study was conducted according to the guidelines of the Declaration of Helsinki and approved by the Committee for Bioethics and Ethics, Medical Department, Aristotle University of Thessaloniki (1.60/21.11.2018). Greek Law of Data Protection was respected through the confidentiality and anonymity of the data.

### 2.3. Measurements

#### 2.3.1. Demographics

Key demographic data were recorded through in-person interviews. There were included questions about sociodemographics, such as age, gender, educational level, diagnosis, and years from diagnosis. The most common systemic comorbidities were also asked for a more comprehensive evaluation of the general health.

#### 2.3.2. Assessment of Visual Function

For clinical evaluation of the residual visual function, distance visual acuity (VA) was measured with the Early Treatment Diabetic Retinopathy Study (ETDRS) chart at 4 meters and best correction was used to determine that participants met the WHO criteria for LV.

#### 2.3.3. Psychological Assessment

*(1) Life Orientation Test (LOT-R)*. The LOT-R [57] is a brief, 10-item questionnaire that evaluates dispositional optimism including three items for optimism (1, 4, and 10), three for pessimism (3, 7, and 9), and four items as fillers that are not scored. Items include statements like “Overall, I expect more good things to happen to me than bad” and “If something can go wrong for me, it will.” According to its developers, it is a unidimensional scale and its internal consistency reliability (Cronbach's alpha coefficient) is promising (0.78), while the Greek version of LOT-R_GR showed also promising psychometric properties (overall alpha = 0.71), revealing two factors (optimism factor, *α* = 0.72; pessimism factor, *α* = 0.66) [58]. The 5-response Likert scale of 0–4 has a range of scores from zero to 24, with higher scores indicating higher levels of optimism. The pessimism items were reverse scored (items 3, 7, and 9).

*(2) Center for Epidemiological Studies-Depression (CES-D)*. The CES-D [59] is a 20-item self-report scale to assess depressive symptoms, covering affective, psychological, and somatic symptoms. The scale was designed to measure the current state and to be responsive to changes over time, so it asks how often each symptom occurred *during* the *past week*. Items include statements like “I felt hopeful about the Future” and “I had crying spells.” According to its developers, the scale has good psychometric properties with a four-factor solution, while the internal consistency reliability (Cronbach's alpha coefficient) is promising (0.80). A strong depressed affect factor (3, 6, 14, 17, and 18), a separate positive affect factor (4, 8, 12, and 16), a somatic negative factor (1, 2, 5, 7, 11, and 20), and a weak fourth factor weighted mainly with the two interpersonal items (dislike and unfriendly) (15, 19), while three items were fillers (9, 10, and 13). The scale has also been validated in the Greek population [60], revealing a three-factor solution: positive affect (items 3, 4, 8, 12, and 16), irritability and interpersonal relationships, depressed affect (items 1, 2, 3, 5, 6, 7, 9, 10, 11, 13, 14, 17, 18, and 20), and somatic complains (items 1, 11, 15, and 19) and a high Cronbach's alpha for the total scale (0.95). The test-retest reliability is satisfactory (Pearson's *r* = 0.45 to 0.95 for individual items and 0.71 for total score). Each answer is scored from zero to 60, with the higher scores indicating more depressive symptomatology. The reversed score is adapted to the scale (4, 8, 12, and 16).

#### 2.3.4. General Vision (GV) and General Health (GH)

Two additional questions were assessed. The questions were designed to capture participants' subjective perceptions regarding their general health and vision. These questions were Likert-type, and they were rated on a 10-point scale (0 representing very poor and 10 excellent), with lower values indicating a higher health burden.

#### 2.3.5. Vision-Related Functionality Questionnaire (LIFE4LVQ)

The 40-item questionnaire [32] evaluates the vision-related functionality of daily life such as shopping, cooking, using public transport, and personal hygiene in two major domains: ability and independence. Specifically, the items include statements about the residual ability to perform vision-targeted activities and the feeling of independence when performing these activities. The 5-Likert response scale gives options to rate ability and level of difficulty from 1 “having no difficulty” to 5 “unable to do because of my vision.” For each item, there is a second question evaluating the feeling of independence, asking patients with LV if they feel that they need some help to perform the activity because of their visual problem, rated from 1 “never” to 5 “always.” Items include statements like “Can you use means of public transport at night or under low luminance conditions?” regarding the ability and “Do you feel you need help in order to use public transportation at night or under low luminance conditions due to your visual impairment?” regarding the independence. According to its developers, LIFE4LVQ reveals a two-factor solution with a good internal consistency reliability (ability, *α* =0.94; dependence, *α* = 0.92), while the overall internal consistency reliability (Cronbach's alpha coefficient) is excellent (around 0.96). Responses have a range of scores from 40 to 202 which is converted to a score between 40 and 100 with high scores representing better vision-related functioning.

### 2.4. Data Analysis

The data were analyzed using IBM SPSS Statistics for Windows, version 27.0. (Armonk, NY: IBM Corp). The sample size calculation was based on the main research question (multivariable regression) for the main prognostic factors, visual acuity, three factors of CES-D (positive affect, irritability and interpersonal relationships, and depressed affect and somatic complaints), and LOT-R (pessimism, optimism), and was conducted using G∗Power (version 3.1.9.7) for the minimum sample size [61]. The alpha level was set to *α* < 0.05 with a power of 0.8. A total of *N* = 46 participants were calculated to identify a large effect size (*f*^2^ = 0.35) [62].

To describe the characteristics of the study, population mean scores and standard deviations were calculated for interval data and percentages for ordinal data. The assumption of normal distribution was investigated for all the variables using the Shapiro-Wilk test. The bivariate correlations between the dependent variable of “LIFE4LVQ” and the variables of the study were calculated using Spearman's *r*. The Mann–Whitney *U* test of statistics was used to explore the difference between two groups, as well as one-way ANOVA (using Tukey correction) for differences among more groups. Variables that showed a significant correlation (*p* < 0.01) as well as variables of interest were taken into account in a regression analysis. The stepwise regression selection method was used to obtain the final multifactorial model. The final variables were included in a correlation matrix to determine their correlations. A *p* value of < 0.05 was considered to indicate statistical significance.

## 3. Results

Descriptive statistics for the measures in this study are presented in Table 1 along with their abbreviations that will be used in the subsequent tables. The majority of participants (71.7%) were above 65 years old, while there was a proportion of participants (28.4%) with age from 18 to 64 years. Most of the participants came from primary or lower secondary education levels with a mean = 10.1 (SD = 4.16) years of education. Moreover, every patient had almost 3 other systemic diseases as comorbidities plus LV condition (*M* = 2.66, SD = 1.66). In the current sample, there was an average of almost 10 years from the LV diagnosis (*M* = 10.32, SD = 11.54) and a mean visual acuity of 54 letters (*M* = 54.04, SD = 14.21). The vast majority of participants were diagnosed with age-related macular degeneration (AMD) (45.3%), another 20.8% with diabetic retinopathy (DR), 11.3% with inherited retinal disease (IDR), 5.7% with glaucoma (G), and another 17.0% suffering from miscellaneous ocular diseases (*M*).

First, correlations were used to give an overview of the relations between the variables (Table 2). The correlation matrix showed a large negative correlation between LOT-R and CES-D (*r* = −0.768, *p* < 0.001), indicating that when the optimistic orientation of life is low, the depressive symptoms are high. GV had a moderate positive relation with VA (*r* = 0 .432, *p* < 0.001) as well as with LIFE4LVQ (*r* = 0.433, *p* < 0.001). Moreover, LIFE4LVQ had a moderate positive correlation with VA (*r* = 0.415, *p* < 0.001). Thus, the way that participants self-evaluated their GV is in concordance with their actual VA measured by clinical tools.

The correlation matrix showed also a moderate negative correlation between GH and systemic comorbidities (*r* = −0.437, *p* < 0.001). As it was expected, when patients experienced more systemic comorbidities, they evaluated their GH as lower. There was also a moderate negative correlation between LIFE4LVQ and CES-D (*r* = −0.423, *p* < 0.001), but not between LIFE4LVQ and LOT-R (*r* = 0.246, *p* > 0.05) revealing that when there is a low functionality in daily living, the depressive symptoms are high. Optimism seems to not correlate with LIFE4LVQ (*r* = 0.246, *p* < 0.001). GH had also a moderate negative correlation with CES-D (*r* = −0.341, *p* < 0.001) as well as a positive correlation with LOT-R (*r* = 0.318, *p* < 0.001). Specifically, when participants evaluated their GH as high, then the depressive symptoms were low. Similarly, when participants evaluated their GH as high, the life orientation of optimism was high too.

Age in years had a moderate positive correlation with systemic comorbidities (*r* = 0.381, *p* < 0.001) as well as with CES (*r* = 0.393, *p* < 0.001) and a negative correlation with LOT-R (*r* = −0.312, *p* < 0.001). This can be explained by the fact that older individuals generally experience more comorbidities than younger. Similarly, older participants had more depressive symptoms than the younger ones, and their perceived optimism was lower than the younger ones. CES-D had a positive moderate correlation with systemic comorbidities (*r* = 0 .331, *p* < 0.001) and a negative correlation with the years from LV diagnosis (*r* = −0.345, *p* < 0.001). As the depressive symptoms scored higher, the systemic comorbidities were higher. Similarly, participants scored with higher optimistic orientation of life (LOT-R), when they had more years from LV diagnosis (*r* = 0.330, *p* < 0.001). Perhaps, as older a patient is and as many years somebody has had the LV diagnosis, they may cope better with this and be more optimistic in life.

Moreover, CES-D had a moderate positive correlation with gender (*r* = 0.305, *p* < 0.001). Particularly, the Mann–Whitney *U* test provided strong evidence of difference, (*U*(1) = 2.199, *p* = 0.028) between gender and CES-D groups. It seems that women had more depressive symptoms scored by CES-D (Mdn = 29.00) than men (Mdn = 14.50) (Table 3).

To explore the relation between the diagnosis and age groups among participants, a one-way ANOVA was conducted, providing strong evidence of a difference (*F*(4) = 7.033, *p* < 0.001), between the means of at least one pair of groups of diagnosis. There was strong evidence (*p* < 0.001) of a difference between the age of AMD (*M* = 78.2, SD = 8.03) and the age of miscellaneous ocular diseases (*M* = 56.1, SD = 19.71). Moreover, the difference between the AMD and IRD was also significant (*p* < 0.002), with the mean age of AMD being higher (*M* = 78.2, SD = 8.03) than the mean age of IRD (*M* = 54.2, SD = 20.10). Generally, it seems that AMD patients are older than IRD and miscellaneous ocular disease groups (Table 4).

Moreover, the relation between the LV diagnosis group and depressive symptoms (CES-D), explored with the Mann–Whitney *U* test, provided a slightly significant difference (*F*(1) = 2.002, *p* = 0.045), within the median of the AMD and non-AMD diagnosis groups. It seems that there is only a slight trend of patients with AMD (Mdn = 27.50) to have more depressive symptoms than patients with LV due to other causes (DR, G, M, and IRD), the non-AMD group (Mdn = 15.00) (Table 5).

All factors of the significant variables (*p* ≤ 0.01) presented on Table 2 as well as factors of scales (variables of interest) were introduced stepwise into a multiple regression analysis to investigate the predicted value of those variables on vision-related functionality as measured with the LIFE4LVQ and how much variance of the LIFE4LVQ score could be explained by those factors. The results of the multiple regression (Table 6) indicated that the final 2-step model explained 31.1% of the variance and that CES-D (positive affect) and VA were significant predictors of the LIFE4LVQ score (*F*(2, 52) = 11.281, *p* < 0.001, *R*^2^ = 0.0311). The symptoms of depression (CES-D positive affect) contributed most to the model as a moderate predictor (*β* = −0.389, *p* = 0.002), followed by visual acuity (*β* = 0.344, *p* = 0.006).

## 4. Discussion

The purpose of this study was to investigate psychological, clinical, and demographic factors that may impact the daily functionality of patients with LV and whether an individual's overall perception of life affects their ability to adapt and cope with LV. The primary findings indicate that the majority of participants were diagnosed with AMD, and those with AMD were older and experienced slightly higher levels of depression compared to those with different diagnoses. Furthermore, older participants exhibited more systemic comorbidities and depressive symptoms, and they were less optimistic. Nevertheless, patients who had been diagnosed with LV for a long time had fewer depressive symptoms and were more optimistic, indicating that strategies to cope with the chronic disease may have been developed. Individuals with LV may develop coping strategies to manage the challenges associated with their disease over time, which is consistent with their overall perception and attitude towards life. Similar studies support that AMD is more prevalent among individuals aged over 75 years old, with the highest incidence occurring after the age of 80 [64]. A literature review indicates that AMD patients are at a higher risk of depression [65], although this remains a controversial issue [66]. Moreover, optimism varies throughout the lifespan and seems to be influenced by age and life events [67, 68]. Optimism tends to be lower in younger adulthood and higher in older adulthood [69–72], with an increase in optimism until middle age and older adulthood before declining in late life [68, 73]. Specifically, a longitudinal study revealed that optimism steadily increases in adults from 50 to about 70 years old. However, in adults over the age of 70, optimism decreases, indicating that it reaches its peak approximately at 68 years old [68]. The findings of the current study suggest that women are more prone to experiencing symptoms of depression than men, and the number of systemic comorbidities is positively correlated with depression. Similar studies are aligned with these results, indicating that depression is more often in women with LV [74] and that depressive symptoms are linked to additional impairments [75]. Other studies highlighted the presence of several chronic health conditions as a risk factor for depression [45, 76] which can lead to greater levels of disability and a decline in health-related QoL [77].

In addition to demographic data, our main results show interesting relationships among emotional state, daily life functionality, personality traits (e.g., optimism), and general health and vision. As expected, when visual acuity (from clinical examination) and perceived general vision (self-assessment) were both low, daily functionality was worse and depressive symptoms were more severe. These findings are consistent with other studies examining the relationship between visual acuity and functionality (using self-reported questionnaires such as the NEI VFQ-25, ADLs, or EQ-5D) or between visual acuity and depression [52, 78–81]. Moreover, according to our results, there is a strong negative correlation between optimism, as a life orientation attitude and depressive symptoms. This may reveal that affected individuals who have previously been pessimistic about life's challenges more often feel depressed, isolated from others, unhappy, and lack the enjoyment of life. Moreover, VA and depressive symptoms (factor of positive affect) were good predictors of the new questionnaire on functionality of daily living (LIFE4LVQ); however, optimism/pessimism as a personality characteristic did not play a role in the prediction model. The factors of “positive affect” (such as loneliness, good, enjoyment, and hopefulness) in the depression scale (CES-D) and VA both predict the functionality of daily living with a variance of 31.1%. In a recent study, depression along with gender and vision coping, also appeared to be strong predictors, explaining 35.2% of the variance in a QoL test [82]. In general, LV is associated with lower levels of psychosocial well-being, manifested by loss of interest and enjoyment in physical activities [83].

The strong negative correlation between dispositional optimism and depressive symptoms of the current findings is consistent with similar research [84], while in another study, low optimism even predicted depression in LV [85]. Therefore, optimism plays an important role in motivating visually impaired individuals to cope with vision loss [86]. Another study—using different measures for personality traits—suggested that neuroticism (a personality trait associated with the likelihood of being tense, insecure, self-conscious, and more prone to experiencing negative emotions such as depression and anxiety [87]) may predispose to a lower level of adjustment to vision loss [53]. These results suggest that the general perception of life plays an important role in the adaptation process and the psychological outcomes. According to the current findings, people with a more pessimistic attitude towards life are more likely to report poorer overall health. On the contrary, a positive attitude towards life tends to promote the maintenance of psychological and emotional resources and brings multiple opportunities to compensate for the changes caused by LV. In a recent longitudinal study, increases in optimism over four years were associated with increases in self-reported health and decreases in chronic illnesses during the same period [68].

In general, psychological factors such as depression and optimism, as well as impaired daily functionality, play an important role in the life of patients with LV. Optimism as a personality trait can help individuals with LV to manage negative emotions more effectively and to accept their visual impairment [88, 89]. The risk of developing depressive symptoms is almost double in patients with LV, revealing the importance of screening for depression and referring patients for rehabilitation or other forms of interventions. A meta-analysis focusing on rehabilitation programs found that they have a positive impact on vision-related QoL, visual functioning, self-esteem, and daily activities. However, no significant difference was found between low vision rehabilitation and inactive comparators in terms of health-related QoL and adaptation to vision loss [25]. Several factors and aspects contribute to the adjustment of visually impaired individuals to their new state life, and the assessment of their psychological and emotional status should become an integral part of a more comprehensive approach to the LV [84].

## 5. Limitations and Future Research

Several limitations of this study must be acknowledged. Data were extracted from a convenience sample from an outpatient clinic. Thus, the results cannot be generalized. Moreover, factors influencing the adjustment to LV, such as the individual's previous life experiences, security, social implications, work status, and the existence of a support network, were not specifically investigated in this study. The role of the general attitude towards life as a coping mechanism in the process of emotional adaptation to LV has not been fully investigated, and this can contribute to future research. However, this is the first thorough investigation of the psychological consequences of LV in Greece, which has the potential to significantly enhance the quality of care provided to these patients by taking their psychological well-being into account. This approach to patients with LV may urge clinicians to pay more attention to the assessment of psychological reactions to provide appropriate counseling in this field. Mental health professionals can draw on patients' positive resources to develop coping strategies and promote engagement in previous activities. Furthermore, this could serve as a useful guide for psychological assessment and support of the unique emotional challenges and needs of these patients. Further and constant investigation in this field is needed to improve the quality of support methods and improve the overall well-being of patients with LV.

## 6. Conclusions

The present study supports that the overall attitude of people with LV is significantly related to psychological status and perception of their general vision and health status along with demographics. It appears that depression and specifically the factor of “positive affect” along with visual acuity may predict levels of functionality in daily life. Although optimism was strongly correlated with fewer depressive symptoms, it was not related to daily functionality. Older age is associated with pessimism, though patients appear to have a more optimistic attitude the longer they have been diagnosed. Although visual acuity is an objective measure of visual impairment, it may not always reflect the special background, needs, and impacts that may vary among affected individuals, so for the same visual acuity, the impact of the loss of vision may vary. Hence, the use of questionnaires may better correlate with the psychological condition and daily functionality, providing insight into the individuals' special needs.

## Figures and Tables

**Table 1:** Clinical and sociodemographic characteristics of the participants.

	*N* (%)		Mean (SD)
Gender		Education (years)	10.09 (4.16)
Male	26 (49.1)		
Female	27 (50.9)		
Age (group)		Age (years)	68.79 (15.95)
18-24	2 (3.8)		
25-34	1 (1.9)		
35-44	1 (1.9)		
45-54	3 (5.7)		
55-64	8 (15.1)		
65-74	15 (28.3)	Systemic comorbidities	2.66 (1.66)
75-84	16 (30.2)		
≥85	7 (13.2)		
Diagnosis		Years from diagnosis	10.32 (11.54)
AMD	24 (45.3)		
DR	11 (20.8)		
IRD	6 (11.3)	Visual acuity (letters)	54.04 (14.21)
G	3 (5.7)		
M	9 (17.0)		

AMD: age-related macular degeneration; DR: diabetic retinopathy; IRD: inherited retinal diseases; G: glaucoma; M: miscellaneous.

**Table 2:** Correlation coefficients (Spearman) for measured variables (*Ν* = 53), *p* > 0.10, *p* > 0.30, and *p* > 0.50 for small, medium, and large effects, respectively [63].

	1	2	3	4	5	6	7	8	9	10	11
(1) Age											
(2) Education	-0.232										
(3) Gender	-0.054	-0.127									
(4) Years from diagnosis	-0.089	0.215	0.085								
(5) Visual acuity	-0.148	0.169	-0.022	0.046							
(6) Systemic comorbidities	0.381^∗∗^	-0.155	-0.004	-0.133	-0.212						
(7) CES-D	0.393^∗^	-0.165	0.305^∗^	-0.345^∗^	-0.073	0.331^∗^					
(8) LOT-R	-0.312^∗^	0.161	-0.107	0.330^∗^	0.170	-0.217	-0.768^∗∗^				
(9) LIFE4LVQ	-0.244	-0.191	0.032	0.224	0.415^∗∗^	-0.252	-0.423^∗∗^	0.246			
(10) GH	-0.195	0.024	-0.192	0.208	0.029	-0.437^∗∗^	-0.341^∗∗^	0.318^∗^	-0.192		
(11) GV	-0.118	-0.025	0.231	0.117	0.432^∗∗^	-0.003	-0.209	0.230	0.433^∗∗^	0.197	

*p* > 0.01^∗∗^, *p* > 0.05^∗^. Abbreviations: CES-D: Center for Epidemiological Studies-Depression; LOT-R: Life Orientation Test; LIFE4LVQ: Life for Low Vision Questionnaire; GH: general health; GV: general vision.

**Table 3:** Median and IQR of CES-D per group of gender conducted by the Mann–Whitney *U* test.

	*N*	Median	CES-D	Test stat.	*p*
			IQR		
Gender					
Women	27	29.00	12.00-33.00		
Men	26	14.50	7.00-19.50		
Total	53	19.00	9.50-32.50	2.199	.028

**Table 4:** Mean differences between and within groups (age∗group of LV diagnosis) conducted by a one-way ANOVA. The Tukey adjustment was applied.

Group of LV diagnosis	*N*	Age		*F*	Sig
		Mean	SD		
DR	11	66.64	9.058		
AMD	24	78.17	8.031		
G	3	69.00	21.377		
IRD	6	54.17	20.104		
M	9	56.11	19.707		
Total	53	68.79	15.954	7.033	<0.001

**Table 5:** Median and IQR of CES per group of sex conducted by the Mann–Whitney *U* test.

Group of LV diagnosis	*N*	Median	CES-D	Test stat. (*H*)	Sig
			IQR		
AMD	24	27.50	14.25-35.75		
Non-AMD	29	15.00	8.50-30.00		
Total	53	19.00	9.50-32.50	2.002	0.045

AMD: age-related macular degeneration.

**Table 6:** Multivariate regression analysis (*n* = 53).

Variable	*β*	*T*	95% CI	*p*
(1) CES-D (positive affect)	-0.389	-3.274	-2.727 to -0.653	0.002^∗∗^
(2) Visual acuity	-0.344	2.894	154 to 0.855	0.006^∗∗^

The above variables correlated significantly with the LIFE4LVQ score. ^∗∗^*p* ≤ 0.01. Variables that did not correlate significantly with LIFE4LVQ were excluded from this model: LOT-R (optimistic), LOT-R (pessimistic), CES-D (depressed affect and somatic complains), and CES-D (irritability and interpersonal relationships).

## Data Availability

The datasets generated during and/or analyzed during the current study are available from the corresponding author on reasonable request.
